# Resistance of wheat genotypes to *Mycosphaerella graminicola* isolates at seedling stage under greenhouse conditions

**DOI:** 10.1002/fsn3.3578

**Published:** 2023-10-09

**Authors:** Tayebeh Bakhshi, Farajollah Shahriari Ahmadi, Mostafa Aghaee Sarbarzeh, Rahim Mehrabi, Alireza Seifi

**Affiliations:** ^1^ Department of Crop Biotechnology and Breeding, Faculty of Agriculture Ferdowsi University of Mashhad Mashhad Iran; ^2^ Seed and Plant Improvement Institute Agricultural Research, Education and Extension Organization (AREEO) Karaj Iran; ^3^ Department of Biotechnology, Faculty of Agriculture Isfahan University of Technology Isfahan Iran

**Keywords:** *M. graminicola*, Septoria leaf blotch (STB), virulence, wheat

## Abstract

One of the most devastating foliar diseases of wheat worldwide is Septoria leaf blotch (STB), caused by *Mycosphaerella graminicola* (asexual stage/Anamorph: *Septoria tritici*) which has been recently intensified in some regions in Iran. In this study, 49 wheat genotypes and 20 wheat differential genotypes were evaluated for their reaction to infection by six isolates of *M. graminicola* collected from infected fields during 2016–2017 at seedling stage under greenhouse conditions. According to the analysis of variance (ANOVA) of leaf pycnidia coverage percentage, a significant difference (*p* < .01) was observed between *M. graminicola* isolates and wheat cultivars. The interaction between genotypes and isolates was also significant (*p* < .01) and the results indicated a specific interaction between genotypes and isolates. The results presented Dezful and West Azerbaijan isolates that were the most virulent with more pathogenesis on differential genotypes. Although 47 of the wheat genotypes were susceptible to all isolates, some genotypes, including Wc‐46,224 (Austria), Wc‐45,425 (Portugal), Wc‐45,565 (Turkey), P.S.No4 (Italy), Dehdasht, M3 Synthetic, KavKaz‐k4500, Arina, Flame, and Riband were resistant to all isolates. In addition, the isolates exhibited different virulence patterns on wheat genotypes. The results of this study revealed high virulence of *M. graminicola* isolates, and Iranian and foreign wheat genotypes, commonly used in the region, presented high susceptibility, and the resistance sources had been identified among genotypes that can be applied in the wheat breeding programs.

## Graphical abstract

In this study, 49 wheat genotypes and 20 wheat differential genotypes were evaluated for their response to the infection by six isolates of M. graminicola isolated from infected fields during 2016–2017 at seedling stage under greenhouse conditions.

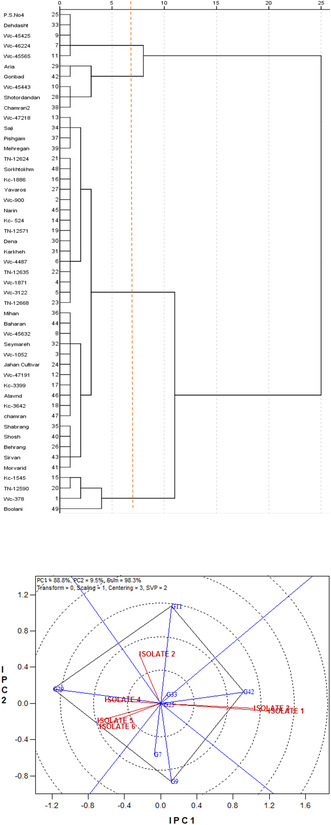

## 1 INTRODUCTION

Septoria leaf blotch (STB) disease is one of wheat's most important foliar diseases, which has been reported in most wheat‐growing areas of the world (Eyal et al., 1987). The pathogen for this disease is *Mycosphaerella graminicola* (*Zymoseptoria tritici*) in the asexual form of Septoria tritici, which reproduces its asexual cycle during growing season under favorable environmental conditions (Kema, Verstappen, et al., 1996). According to recent phylogenetic studies, the name *Zymoseptoria* has been suggested for the pathogen (Quaedvlieg et al., 2011). Wheat STB was first reported by Desmaziers (1842) from France and later it was reported from other parts of the world including Europe, Africa, Asia, North America, Central and South America, and Australia (Shearer & Wilcoxson, 1978). The sexual form of this fungus (*M. Graminicola*) was first identified by Sanderson (1972) in New Zealand and later in Australia, Brazil, the Netherlands, the United Kingdom (Eyal et al., 1987), and Canada (Hoorne et al., 2002).

Wheat STB disease in Iran was first reported by Petrak which was then sporadically and negligibly on wheat (Dadrezaie et al., 2003). The disease has gradually become more important and expanded in Iran with beginning of cultivation of modified CYMMYT genotypes (Khelghatibana et al., 2004; Rajaie et al., 2004). The extension of STB will be much more intensive with the development of rust‐resistant dwarf genotypes as well as with increased nitrogen fertilizer use and disease loss will be intensified if infection occurs prior to spike emergence (Eyal, 1999). The fungi caused the disease result in unexpected and very serious epidemics on susceptible genotypes in favorable environment, and significantly reduce yield quality and quantity, which in some cases exceeds up to 50% losses (Eyal et al., 1987).

Cultivating method control such as spraying fungicides and using resistant genotypes are recommended to control this disease. Given the inefficiency of cultivating methods in the effective control of the disease, resistance of fungal isolates to fungicides, and the costs and pollution caused by the application of toxic chemicals, the use of resistant genotypes are considered one of the most cost‐effective and best methods to control the disease (Eyal, 1999; Eyal et al., 1987). Physiologic specialization of the gene‐for‐gene was demonstrated in this pathosystem (Brading et al., 2002) several years ago and 22 resistance genes (*Stb*) were mapped to STB in different wheat genotypes (Arraiano et al., 2001, 2003, 2007; Adhikari, Wallwork, & Goodwin, 2004; Brading et al., 2002; Chartrain, Berry, & Brown, 2005; Chartrain, Joaquim, et al., 2005; Chartrain, Brading, et al., 2005; Chartrain et al., 2009; Cowling, 2006; McCartney et al., 2003; Tabib Ghaffary et al., 2011, 2012; Yang et al., 2018). Previous studies proved that the growth of fungal biomass terminates in the resistant genotype with *Stb* genes after 12–15 days (Habibi et al., 2014). Specific resistance to *M. graminicola* single isolates was identified in bread wheat genotypes (Abrinbana et al., 2012) and local tetraploid wheat subspecies (Ghaneie et al., 2012). Unavailable information regarding pathogen virulence and specific resistance to different isolates makes it difficult to research and identify how genetically control resistance in resistant genotypes and their utilization in breeding programs for resistance to STB. A study of the genetic structure of *M. graminicola* using molecular markers revealed that high genetic differentiation and low levels of gene flow were observed among the populations of this fungus in Ardebil, Golestan, Khuzestan, Fars, and East Azerbaijan provinces (Abrinbana et al., 2010). The transmission of various genes, including virulence and avirulence genes, are restricted among the fungi populations in these areas under these circumstances. Accordingly, it is proposed that the response of the genotypes and lines used in each of these areas are examined with isolates of the same region to identify sources of effective resistance (Abrinbana et al., 2010). The purpose of this study is to aim to compare the resistance of various wheat genotypes and identify those with the highest level of resistance to STB for use in wheat breeding programs.

## 2 MATERIALS AND METHODS

### 2.1 Plant material

Seeds used in this study include three types of genetic materials including 13 bread wheat cultivars (*Triticum aestivum* L., 2*n* = 6*x* = 42), 25 durum wheat genotypes (*Triticum durum* L., 2*n* = 4*x* = 28) including Iranian and landrace from other countries, eight accessions from eight countries: Austria, Afghanistan, Portugal, France, Turkey, Argentina, Bulgaria, and Italy, ten durum wheat cultivars, one susceptible control (Boolani), and 20 wheat differential genotypes including two susceptible control (Taichung 29 and Obelisk) (Tables 1 and 2). Seeds of 49 genotypes were received from the gene bank of Cereal Research Department of Seed and Plant Research Improvement Institute, Karaj, Iran.

**TABLE 1:** List of Durum and Hexaploid wheat genotypes used in this study.

No.	Wheat genotypes	Provinces	Origin	Type
1	Wc‐378	Ardebil	Iran	LA
2	Wc‐900	Golestan	Iran	LA
3	Wc‐1052	Lorestan	Iran	LA
4	Wc‐1871	West Azerbaijan	Iran	LA
5	Wc‐3122	Khorasan	Iran	LA
6	Wc‐4487	Lorestan	Iran	LA
7	Wc‐46,224	Austria	Austria	LA
8	Wc‐45,632	Afghanistan	Afghanistan	LA
9	Wc‐45,425	Portugal	Portugal	LA
10	Wc‐45,443	France	France	LA
11	Wc‐45,565	Turkey	Turkey	LA
12	Wc‐47,191	Argentina	Argentina	LA
13	Wc‐47,218	Bulgaria	Bulgaria	LA
14	Kc‐ 524	Khuzestan	Iran	LA
15	Kc‐1545	Kermanshah	Iran	LA
16	Kc‐1886	Isfahan	Iran	LA
17	Kc‐3399	Khorasan	Iran	LA
18	Kc‐3642	Kermanshah	Iran	LA
19	TN‐12571	Kohgiluyeh and Boyer‐Ahmad	Iran	LA
20	TN‐12590	Sistan and Baluchestan	Iran	LA
21	TN‐12624	West Azerbaijan	Iran	LA
22	TN‐12635	Hamedan	Iran	LA
23	TN‐12668	Khuzestan	Iran	LA
24	Jahan Cultivar		Iran	LA
25	P.S.No4	Italy	Italy	LA
26	Behrang		Iran	CV
27	Yavaros		Iran	CV
28	Shotordandan		Iran	CV
29	Arya			Iran
30	Dena			Iran
31	Karkheh			Iran
32	Seymareh			Iran
33	Dehdasht			Iran
34	Saji			Iran
35	Shabrang			Iran
36	Mihan			Iran
37	Pishgam			Iran
38	Chamran2			Iran
39	Mehregan			Iran
40	Shosh			Iran
41	Morvarid			Iran
42	Gonbad			Iran
43	Sirvan			Iran
44	Baharan			Iran
45	Narin			Iran
46	Alavnd			Iran
47	Chamran			Iran
48	Sorkhtokhm			Iran
49	Boolani			

Abbreviations: CV, cultivar; LA, Landrace.

**TABLE 2:** List of wheat differentials used in this study and their resistance genes (*Stb*).

No.	Wheat genotypes	Origin	Stb genes	References
50	Cs Synthetic 6X	China/USA	Stb5	Arraiano et al. (2001)
51	Oasis	USA	Stb1	Adhikari, Yang, et al. (2004)
52	Kavkaz‐K4500	CYMMIT	Stb10, Stb12 (Stb6, Stb7)	Chartrain, Berry, and Brown (2005)
53	Arina	Switzerland	Stb15 (Stb6)	Arraiano et al. (2007), Chartrain, Brading, and Brown (2005)
54	Riband	United Kingdom	Stb15	Arraiano et al. (2007)
55	Flame	United Kingdom	Stb6	Brading et al. (2002)
56	M3 synthetic (W‐7976)	USA	Stb16, Stb17	Tabib Ghaffary et al. (2012)
57	TE9111	Portugal	Stb11 (Stb6, Stb7)	Chartrain, Joaquim, et al. (2005)
58	Estanzuela Federal	Uruguay	Stb7	McCartney et al. (2003)
59	Balance	France	Stb18	Tabib Ghaffary et al. (2011)
60	M6 synthetic (W‐7984)	USA	Stb8	Adhikari et al. (2003)
61	Courtot	France	Stb9	Chartrain et al. (2009)
62	Israel 493	Israel	Stb3 (Stb6)	Adhikari, Wallwork, and Goodwin (2004), Chartrain, Brading, and Brown (2005)
63	Veranopolis	Brazil	Stb4 (Stb6)	Adhikari, Wallwork, and Goodwin (2004), Chartrain, Brading, and Brown (2005)
64	Tadinia	USA	Stb4 (Stb6)	Adhikari, Cavaletto, et al. (2004), Chartrain, Brading, and Brown (2005), Somasco et al. (1996)
65	Salamouni	Canada	Stb13, Stb14	Cowling (2006)
66	Shafir	Israel	Stb6	Brading et al. (2002)
67	Bulgaria 88	Bulgaria	Stb1	Arraiano et al. (2001)
68	Taichung29	Japan	Susceptible control	–
69	Obelisk	Netherland	Susceptible control	–

### 2.2 Collection of infected plant samples

The naturally infected leaves with Septoria leaf blotch from wheat fields in different regions of Iran were collected in 2016–2017 cropping season, the samples were then transferred to the laboratory, and subjected to fungal isolation, for which 1–2 disks per each leaf containing pycnidia were cut as sample. The samples were superficially disinfected with 2% sodium hypochlorite. The leaf disks were placed on wet filter paper in sterile petri dishes and kept at 20°C for 24 h. The oozes from the pycnidia ostiole were transferred onto potato‐dextrose‐agar (PDA; potato 200 g/L, dextrose 20 g/L, and agar 15 g/L) plates under a stereoscopic microscope with a sterile fine needle. The plates were then kept to allow colonies to grow. The samples were purified by drawing sketches on laboratory loop impregnated with spore suspension and the samples were then subcultured on PDA medium as pure fungal culture. Seedlings were prepared by sowing wheat seeds with five to seven seeds in each plastic pot containing a mixture of peat moss and soil in a ratio of 1:1 in three replications. In order to prepare the suspension required for seedling inoculation, the liquid medium of yeast‐glucose extract (YGM; 30 g/L glucose +10 g/L yeast extract + distilled water) was infected with segments of colony grown on PDA medium and placed on a shaker at 17°C for 5 days. The prepared spore suspension was centrifuged at 3000 rpm for 5 min and then the suspension of yeast spores deposited was prepared in sterile water with a concentration of 10 million spores per mL (10^6^ Spor/mL).

### 2.3 Zymoseptoria tritici isolates

Due to their specific interactions with some wheat genotypes (Tabib Ghaffary et al., 2012), six *Z. tritici* isolates (five isolates from Iran and one isolate from Algeria) were selected to be used in this study from different origins (Table 3).

**TABLE 3:** List of origins of *Zymoseptoria tritici* isolates used in this study.

Isolates	Code	Province	Country
1	RM 251	Guelma	Algeria
2	RM 155	Dezful	Iran
3	RM 5	Fars	Iran
4	RM 22	Khuzestan	Iran
5	RM 183	Ardebil	Iran
6	RM 230	West Azerbaijan	Iran

### 2.4 Evaluation of isolates' virulence using wheat cultivars in greenhouse

The virulence of six *M. graminicola* isolates was tested using 69 wheat cultivars and three control susceptible cultivars to Septoria leaf blotch disease (Bolani) in a completely randomized design with three replications in greenhouse conditions. Ten seeds of each genotype were sowed in 10‐cm pots containing a mixture of field soil and peat moss in a ratio of 1:1, the seedlings were inoculated at the one‐leaf stage (approximately 9 days after sowing when the first leaf completely expanded and the second leaf appeared) separately with fungal spore suspension with a concentration of 10^7^ Spore/mL for each isolate by spraying until the spore suspension flowed from the leaf surface. The test was applied in greenhouse according to Tabib Ghaffary et al. (2012) and Abrinbana et al. (2012) with small modifications.

The inoculated seedlings were kept for 24 h in the dark, at a temperature of 18°C and a high relative saturated humidity (> 85%), and then transferred to greenhouse with a photoperiod of 16‐h light with an intensity of 12,000 lux and 8‐h darkness with above‐mentioned temperature and humidity. The seedling responses were analyzed 21 days after inoculation by measuring the percentage of leaf area with necrotic lesions bearing pycnidia (Kema, Verstappen, Todorova, & Waalwijk, 1996; Kema et al., 1996) and McCartney et al. Scale (McCartney et al., 2003).

Analysis of variance of data obtained from measuring percentage of pycnidia coverage (PC) and necrosis level (NL) of leaves was performed after standardization using SPSS and Excel software, categorization of isolates and wheat cultivars based on average percentage of pycnidia coverage, and percentage of necrosis in leaf area using cluster analysis in the Ward and GGE Biplot approach.

## 3 RESULTS AND DISCUSSION

Characteristics of wheat genotypes, differential genotypes, and *Z. tritici* isolates used in this study are presented in Tables 1, 2, and 3, respectively.

### 3.1 Response of wheat genotypes and comparison of virulence of *M. graminicola* isolates based on pycnidia coverage percentage

Results of analysis of variance (ANOVA) indicated significant differences (*p* < .01) between cultivars and isolates (Table 4). The analysis also revealed significant differences (*p* < .01) in interaction between genotypes and isolates, indicating a specific interaction between the studied genotypes and isolates. This may show the genetic differences between the host genotypes for resistance to the pathogen. According to the results of this test, the mean of infection was 0% and the interactions that were not significantly different from the mean of 0% were considered as resistance.

**TABLE 4:** Results of combined variance analysis of wheat genotypes pycnidia and necrosis studied isolate of Septoria.

Source of variation	df	Mean squares	
		*p*	*n*
Isolate	5	1326.29**	167.82**
Error(a)	12	817.36	32.18
Genotype	68	5188.43**	957.51**
Genotype×race interaction	340	348.65**	393.32**
Error(b)	816	112.30	134.13
Coefficient of variation (%)		9.12	7.86

ns, *, **: Nonsignificant, Significant at 5% and 1% probability levels, respectively.

The responses of 49 wheat genotypes to six *M. graminicola* isolates were investigated in this study, among which the landraces genotypes of Wc‐46,224 (Austria), Wc‐45,425 (Portugal), Wc‐45,565 (Turkey), P.S.No4 (Italy), Dehdasht (Iranian durum cultivar) were the most resistant genotypes with resistance to six isolate (Figure 3), suggesting that these genotypes also possess resistance genes (*Stb10*, *Stb12*, *Stb15*, *Stb16*, and *stb17*) effective against a limited number of *Z. tritici* isolates, followed by the most resistant wheat Aria (durum wheat cultivar)–Gonbad (bread wheat cultivar) and Shotordandan (a local durum wheat cultivar) –Chamran2 (bread wheat cultivar)–Wc‐45,443 (an accession from France origin) and Behrang (durum wheat cultivar) were resistant to three isolates, two isolates, and one isolate, respectively. Although the above genotypes presented isolate‐specific resistance, the results of this study indicated that most of the wheat genotypes in this study, especially wheat genotypes of Wc‐1871 (East Azerbaijan), Wc‐3122 (Khorasan), TN‐12624 (West Azerbaijan), TN‐12635 (Hamedan), Narin, and Sorkhtokhm with the highest mean disease severity were susceptible to STB. The results indicated almost 77.5% of the studied genotypes (38 genotypes) were susceptible to all fungal isolates, 10.2% presented highly resistant reaction (five genotypes), and 12.2% isolate‐specific resistance (six genotypes) was identified in the other genotypes (Table 5).

**TABLE 5:** Percentage of frequency of resistance and susceptible genotypes based on pycnidia coverage.

Isolate	Pycnidia /necrosis	Cultivar/differential	Resistant reaction		Susceptible reaction
			Number of cultivars	Percentage (%)	Percentage (%)
1	Pycnidia	Cultivar	7, 9, 10, 11, 25, 26, 28, 33, 38, 42	20.4	79.5
		Differential	51, 52, 53, 54, 55, 56, 57, 63, 64, 65, 67	55	45
2	Pycnidia	Cultivar	7, 9, 10, 11, 25, 28, 33, 38	16.3	83.6
		Differential	52, 53, 54, 55, 56, 57	30	70
3	Pycnidia	Cultivar	7, 9, 11, 25, 33, 42	12.2	87.7
		Differential	51, 52, 53, 54, 55, 56, 65	35	65
4	Pycnidia	Cultivar	7, 9, 11, 25, 29, 33, 42	14.2	85.7
		Differential	51, 52, 53, 54, 55, 56, 63, 65	40	60
5	Pycnidia	Cultivar	7, 9, 11, 25, 29, 33	12.2	87.7
		Differential	51, 52, 53, 54, 55, 56, 65	35	65
6	Pycnidia	Cultivar	7, 9, 11, 25, 29, 33	12.2	87.7
		Differential	52, 53, 54, 55, 56, 65	30	70

The susceptible control genotypes (Boolani, Taichung 29, and Obelisk) were susceptible to all isolates. Evaluation of the virulence pattern of *M. graminicola* isolates on wheat genotypes revealed that these isolates were different in terms of virulence and none of them performed virulence/avirulence in all genotypes (Table 4).

Among the isolates, RM 5, RM 183, and RM 230 with 87.7% virulence on 43 genotypes and RM 251 with 79.5% virulence on 10 genotypes had the highest and lowest virulence isolates, respectively (Table 5).

As described by Brown et al. (2001), the isolate aggressiveness was measured for each wheat genotype on the basis of the mean disease severity by ignoring data for specific interactions. The most and the least aggressive isolates were RM230 and RM251, which presented the highest mean disease severity (40.1%) and the lowest mean disease severity (37%), respectively (Table 4).

To understand the pathogenicity and resistance in pathosystem of *M. graminicola* and wheat, it should be considered that wheat genotypes are continuously exposed to completely diverse populations of pathogens and the abundance of pathogenic isolates with specific pathogenicity, which are capable of adjusting and establishing different genotypes of wheat, and are gradually increasing. Investigation of pathogenicity differences of 56 *M. graminicola* isolates collected from seven provinces of Iran revealed that there are significant differences among the isolates in terms of invasive power (Bashiri et al., 2006).

### 3.2 *Z. tritici* isolates’ aggressiveness and *Stb* genes’ efficacy against isolates

Different virulence patterns on the *Stb* differentials were observed in six isolates, indicating the effective resistance genes to a limited number of *Z. tritici* isolates in these genotypes.

The results presented that Kavkaz‐k4500 (possessing *Stb10*, *Stb12*, *Stb6*, and *Stb7*), Arina (possessing *Stb15* and *Stb6*), M3 synthetic (W‐7976) (*Stb16* and *Stb17*), Flame (possessing *Stb6*), and Riband (possessing *Stb15*) were resistant to all isolates. Salamouni possesses *Stb13* and *Stb14* resisted all isolates except RM 155. Oasis and Bulgaria possess *Stb1*, Oasis showed resistance to four isolates (RM 251, RM 6, RM 22, and RM 183), while Bulgaria showed resistance responses to one (RM 251) isolate. Veranopolis (possessing *Stb2* and *Stb6*) and TE9111 (possessing *Stb11*, *Stb6*, and *Stb7*) genotypes were resistant to two isolates ((RM 251, RM22) and (RM 251, RM 155)), respectively. Tadinia possesses *Stb4* and *Stb6* which were resistant to RM 251 isolate, and also Bulgaria 88 possesses *Stb1* which was resistant to RM 251. The rest of the differential genotypes were susceptible to the isolates. Among *Stb* genes, *Stb10*, *Stb12*, *Stb15*, *Stb16*, and *Stb17* genes were the most effective resistance genes that presented resistance to all isolates. The six isolates used in this study were different in aggressive and mean disease severity on 20 differential wheat genotypes. RM 155 and RM 230 high virulence isolates were pathogenic on 14 differential genotypes and RM 251 isolates were the least virulent isolates and were pathogenic on nine differential genotypes. In terms of aggression, RM 155 isolate with the highest mean disease severity (44.1%) was the most aggressive isolate. The RM 251 isolate with the lowest mean disease severity (27.1%) had the lowest invasive power (or was the lowest aggressive isolate).

### 3.3 Cluster analysis

**FIGURE 1:**
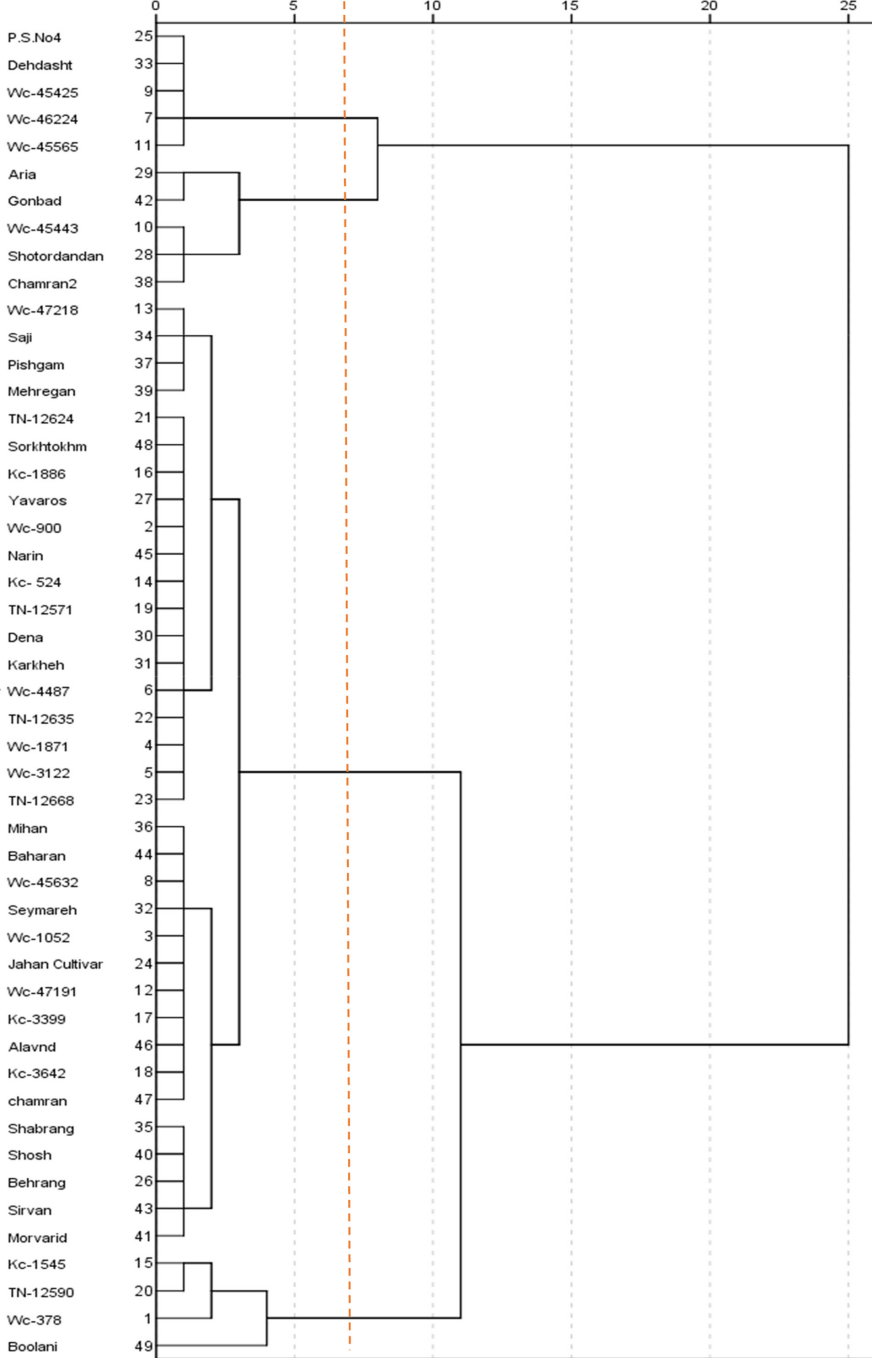
Cluster analysis of 49 durum and hexaploid wheat genotypes based on their mean disease severities to each isolate.

Cluster analysis results of the genotypes based on the mean pycnidia coverage percentage of leaf area grouped them into four separate clusters in response to isolates (Figure 1).

Cluster I containing five genotypes including four landraces (Wc‐46,224, Wc‐45,425, Wc‐45,565, and P.S.No4) and one Iranian durum wheat cultivar (Dehdasht) showed high level of resistance to six isolates and mean disease severity data ranged from about 0% to 9.1% (Figure 1 and Table 5).

In cluster II, the isolate‐specific resistance was identified in one landrace, two durum wheat cultivars, and two bread wheat cultivars including Wc‐45,443 (France), Chamran2, and Shotordandan which were resistant to two isolates (RM 251 and RM 155), while Aria and Gonbad showed resistance responses to three isolates, respectively, RM 22, RM 183, and RM 230 and RM 251, RM 5, and RM 22 (Figure 1 and Table 5).

In clusters III and IV, 39 genotypes (27 landraces and Iranian durum wheat cultivars, 10 bread wheat cultivars, and susceptible control) with low to high susceptible reactions were grouped with control showing the mean infection of 37.6% to about 85.5% (Figure 1 and Table 5).

The results of cluster analysis of differential genotypes also revealed that these genotypes can be divided into resistant and susceptible groups in response to *M. graminicola* isolates.

**FIGURE 2:**
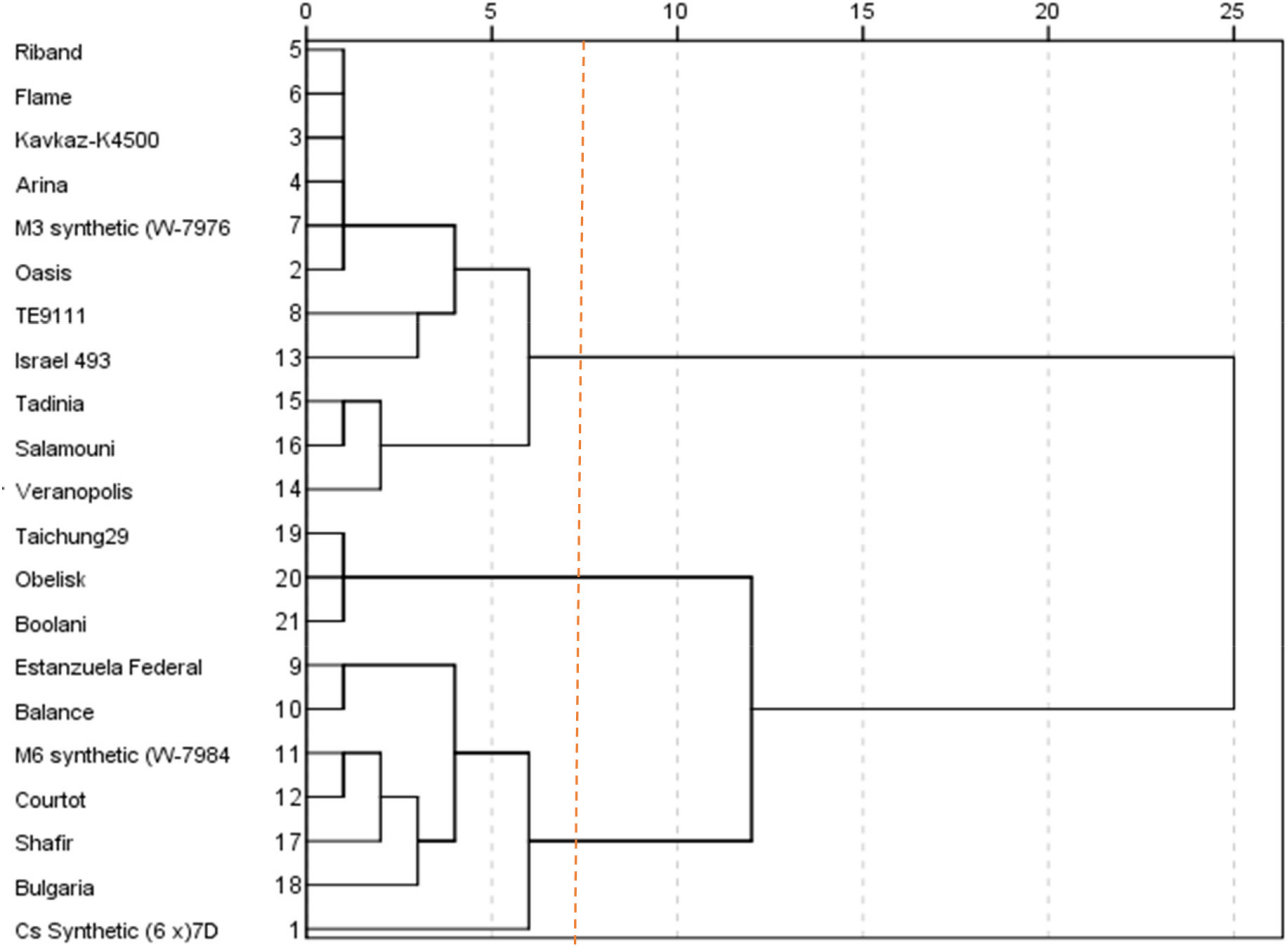
Cluster analysis of wheat differential cultivars based on their mean disease severities to each isolate.

Cluster I includes two categories of genotypes, the first group (Kavkaz‐k4500, Arina, Riband, Flame, M3 Synthetic) presented high resistance to all isolates, ranging from 0% to 5.3% (Figure 2 and Table 5).

The second group containing five genotypes including Oasis, Te9111, Tadinia, Salamouni, and Veranopolis in which the isolate‐specific resistance was identified in cluster I, respectively, were resistant to RM 251, RM 5, RM 22, and RM 183; RM 251 and RM 155; RM 251; RM 251, RM 5, RM 22, RM 183, and RM 230; and RM 251 and RM 22 isolates (Figure 2 and Table 5).

Clusters II and III contained control susceptible genotypes (Boolani, Taichung29, and Obelisk) and seven genotypes that indicated highly susceptible pattern to all isolates with a range of 44.2–66.3% (Figure 2 and Table 5).

### 3.4 Analysis of GGE biplot of genotypes compared to isolates

#### 3.4.1 The superior genotypes against Septoria tritici blotch caused by *M. graminicola* isolates

**FIGURE 3:**
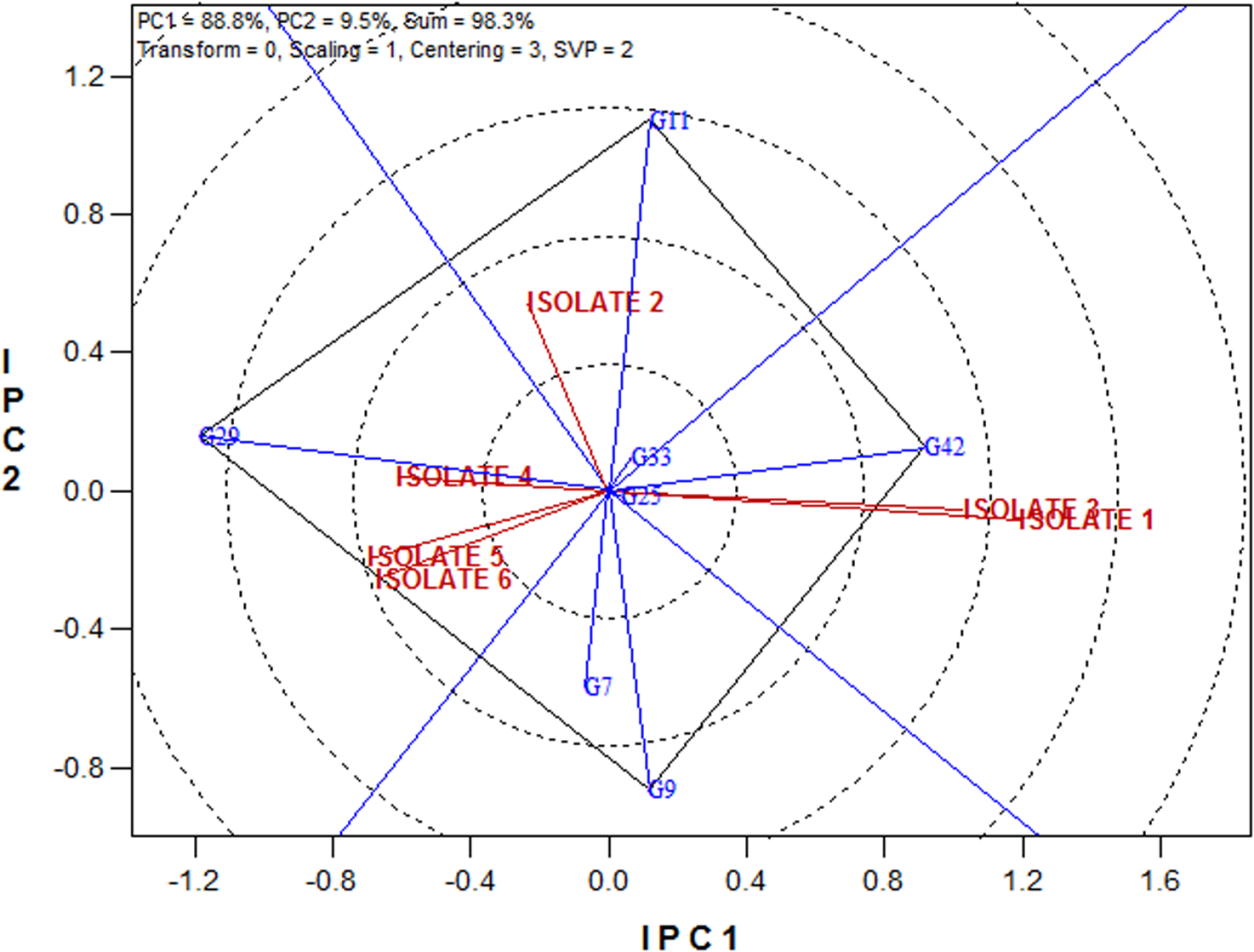
The superior genotypes against Septoria tritici blotch caused by *M. graminicola* isolates.

In Figure 3, the superior genotypes are placed on the top of polyhedron relative to each isolate based on mean disease severity data. Superior genotypes, in terms of resistance components, measured against Septoria isolates were genotype 11 for isolate 2, genotype 42 for isolates 1 and 3, and genotype 29 for isolates 4, 5, and 6 (Figure 3).

#### 3.4.2 Relationships among studied Septoria isolates

**FIGURE 4:**
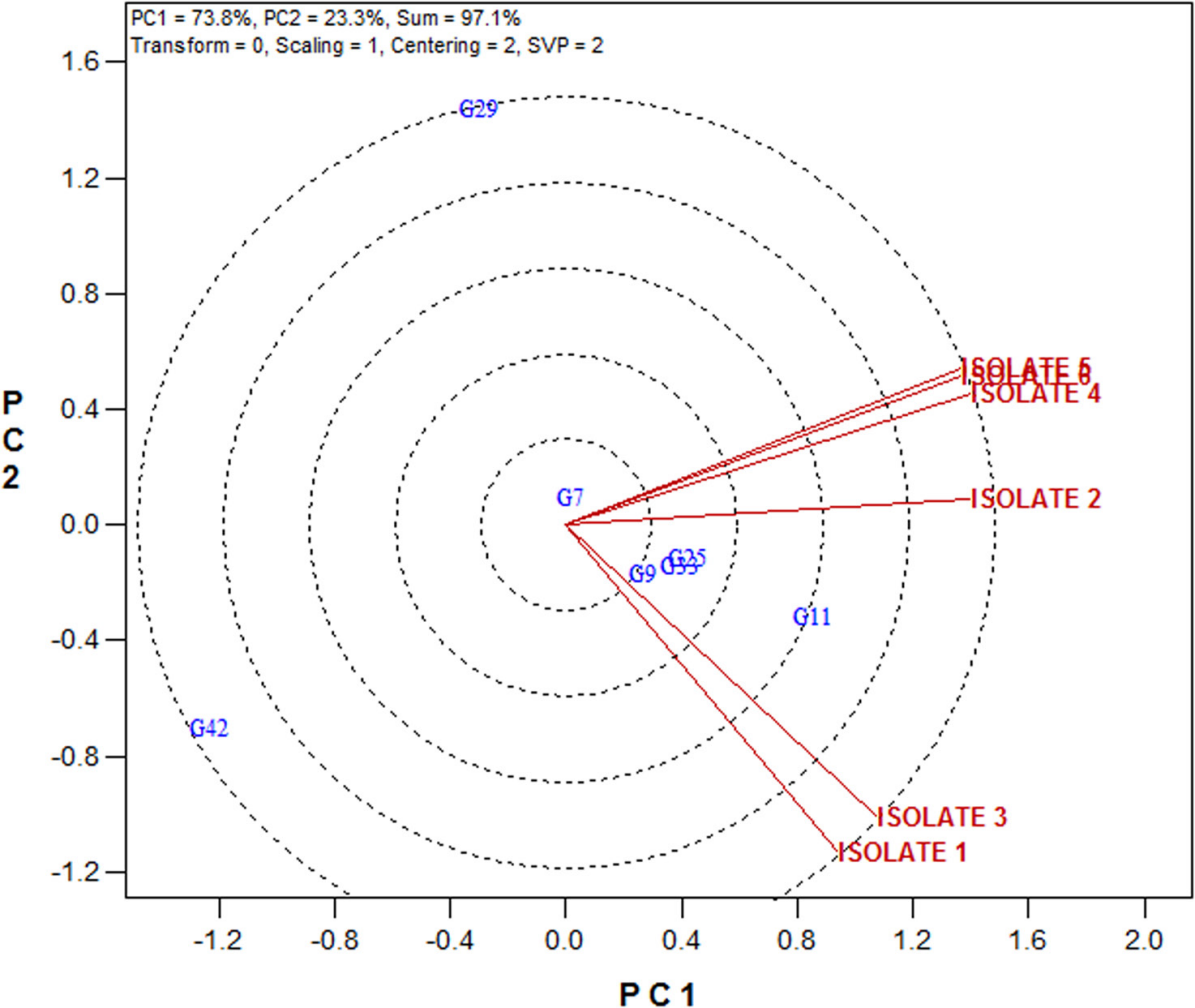
Relationships among studied Septoria isolates.

The relationships among isolates can be seen in Figure 4. A close relationship was observed among isolates 1 and 3 with isolates 4, 5, and 6; in other words, they have more similarities in terms of virulence pattern. Isolate 2 is more distinguished from other isolates, so its virulence pattern is different from other isolates (Figure 4).

### 3.5 Relationships between the superior wheat genotypes and Septoria isolates

**FIGURE 5:**
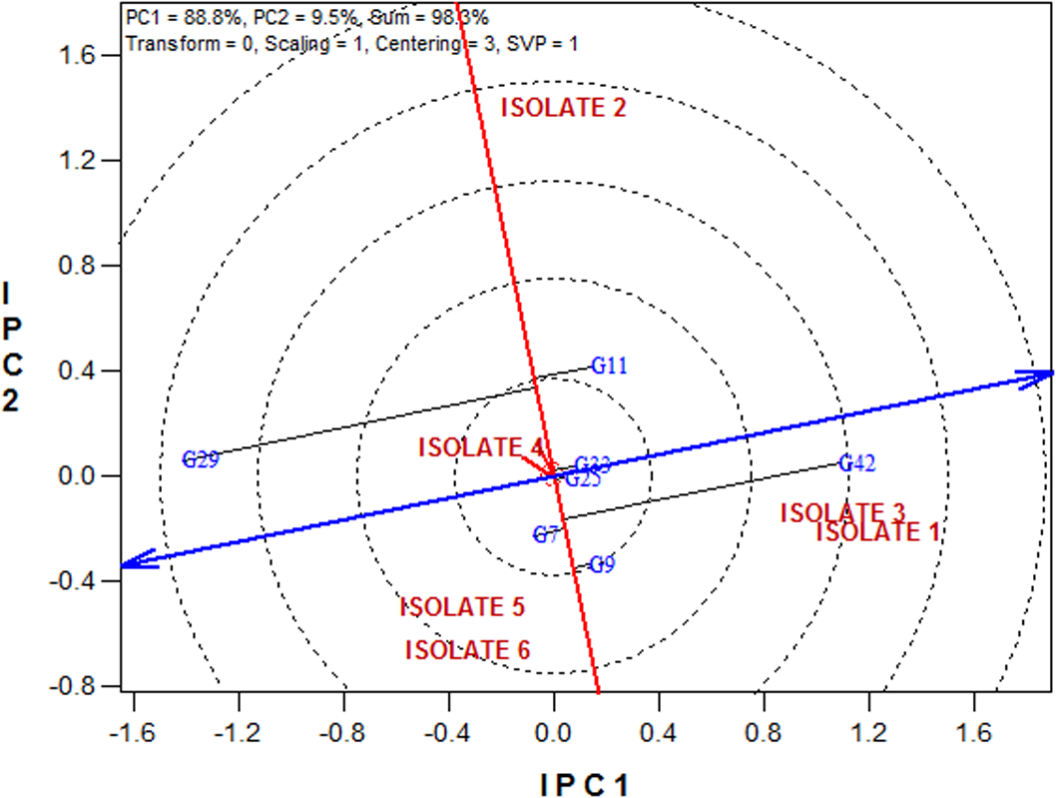
Relationships between the superior wheat genotypes and Septoria isolates.

The relationships of the identified superior genotypes were revealed based on mean disease severity data to the studied isolates in Figure 5. Therefore, genotypes 11, 33, 25, 9, and 7 were resistant genotypes with the highest resistance to all isolates. Genotype 42 for isolates 1 and 3, and genotype 29 for isolates 4, 5, and 6 had acceptable specific resistance (Figure 5).

## 4 DISCUSSION

Yield losses caused by wheat STB are increasing in recent decades, plant pathologists and wheat breeders have further focused on the disease and have studied its various aspects such as genetic diversity, virulence pathogen, and host resistance. The use of resistant genotypes not only provides the best and most effective way to control the disease economically and environmentally but also it can contribute to avoid the use of chemical‐based fungicides (Eyal, 1999).

According to the results of studies conducted in Iran (Haghdel & Banihashemi, 2003; Khelghatibana et al., 2004; Kia et al., 2006) and the incidence of disease epidemics in some provinces of the country, most common wheat genotypes used in Iran appeared to be susceptible to this disease, and serious consideration to breeding resistant varieties of the disease requires study of virulence pathogenicity in different regions, evaluation of resistance of various wheat genotypes against isolates of infected areas, identification of resistant genotypes, and finally study of their genetic resistance. The present study was conducted in this regard and single isolate of *M. graminicola* was used to evaluate the response of resistant genotypes. The results of this study revealed the presence of specific resistance in the studied wheat genotypes and the physiological specialization of the isolates, which are consistent with the findings of other studies (Eyal et al., 1985; Grieger et al., 2005; Kema, Annane, et al., 1996).

In Makhdoomi and his co‐worker's study, Aria (durum cultivar) was resistant to all isolates (Makhdoomi et al., 2011), while in other studies, Shotordandan (durum cultivar) and Dehdasht (bread cultivar) were the most resistant cultivars (Dalvand et al., 2016; Davari et al., 2012).

These results are consistent with the findings of Eyal et al. (1973) who first reported the presence of virulence differences in *M. graminicola* isolates and found virulent specific genes on some genotypes by assessing the virulence of 97 isolates on 35 wheat and triticale genotypes (Eyal et al., 1985). Razavi and Hughes (2003) suggested that there is a significant difference in virulence and invasive strength among 90 isolates collected from a field.

In this research, Wc‐46,224, Wc‐45,425, Wc‐45,565, P.S.No4, Dehdasht, KavKaz‐k4500, Arina, Riband, Flame, and M3 Synthetic were the most resistant genotypes. This indicates that *Stb10*, *Stb12*, *Stb15*, *Stb16*, and *Stb17* are found to be in the ten resistant genotypes in combination or individually.

The mean total infection of the genotypes such as Aria, Oasis, TE9111, and Salamouni was low, due to the large number of isolate‐specific resistance in these genotypes. Therefore, the studied genotypes presented no moderate resistance to the isolates. These results indicated that most of these genotypes are susceptible to wheat STB, which could justify the disease's outbreak and epidemic in this area. Although bread hexaploid wheat and tetraploid durum wheat are the most important hosts of *M. graminicola*, some studies have shown that host specificity at the host species level was also present among isolates of this pathogen, so isolates collected from durum wheat are nonpathogenic on bread wheat (Kema, Annane, et al., 1996; Van Ginkel & Schafren, 1988). In regions or countries where one of these wheat genotypes was predominantly cultivated, the fungal isolates of those areas were adjusted to the predominant wheat, and specialization at the host species level occurs as an important trait (Eyal, 1999).

A few number of isolates used in this study were different in the virulence pattern of isolates and probably reflect high genetic diversity in the fungal populations in the region. These results were similar to the findings of most of the research conducted in these areas (Abrinbana et al., 2012; Eyal et al., 1973; Eyal et al., 1985; Kema, Annane, et al., 1996; Makhdoomi et al., 2015; Mehrabi et al., 2015), these studies used different isolates through the world. There has been limited information available on the virulence diversity of *M. graminicola* isolates in a specific region or even a specific country to this date. However, in a study using isolates collected from Manitoba and Saskatchewan in Canada, the low virulence diversity of the isolates was reported for these fungi populations in the region (Grieger et al., 2005). Although disease scaling has been used as one of the methods of evaluation of wheat STB in some cases (Grieger et al., 2005; Mergoum et al., 2007; Rosielle, 1972), the expression of single‐gene or vertical resistance to the disease has not been always decisive and qualitative and, in some cases, this type of resistance also occurs quantitatively. So, unlike some diseases such as wheat powdery mildew, resistance to STB has been assessed quantitatively, usually by measuring the percentage of host leaf area covered by lesions bearing pycnidia (Brown et al., 2001; Chartrain, Brading, Makepeace, & Brown, 2004). In this disease, the level of infection was continuous, ranging from complete immunized (without pycnidia coverage) to complete susceptible with 90% leaf area infection, which symptoms vary depending on the studied wheat isolates and genotypes (Chartrain, Brading, Makepeace, & Brown, 2004; Eyal, 1999). That was why the use of qualitative methods was not very accurate and it was recommended to evaluate the disease quantitatively by calculating the percentage of host leaf covered by pycnidia and identifying the specific isolate–host interaction in this pathosystem by statistical methods. Accordingly, in this study, the percentage of leaf area covered by lesions bearing pycnidia was considered as a criterion for disease evaluation, and race‐specific resistance was determined by statistical method. A large number of specific interactions was identified by this method, in some of which the resistance was not decisive and qualitative as up to 5% infection was observed in some cases.

According to the results of this study, it seems that the isolates of *M. graminicola* had high genetic diversity and virulence, which makes it difficult to efficiently use resistant genotypes and breeding for resistance to wheat STB in some cases. Most of the common genotypes in the region, especially the durum wheat genotypes studied in this study, were susceptible to the disease, and some of them had resistance genes that were not effective against most isolates in the region. However, among these genotypes, resistance sources were identified that could be used in wheat breeding programs in the region. This requires the study of resistance genetics and identification of the genes that cause resistance in these genotypes. Furthermore, it is advisable to evaluate the response of other genotypes and lines to identify more effective resistance sources and to produce genotypes with wider spectrum resistance by pyramidization of the effective genes. Pyramidization of resistance genes may not be effective in the long run due to the high genetic diversity of the pathogenic population in this region and the potential for sexual reproduction of the fungus. Moderate resistance should be used along with single genes in order to achieve stable resistance and to prevent rapid breakage of resistance. The genotypes that had been studied in this research did not show a relative resistance, but genotypes with this resistance may be identified by studying other genotypes and lines, or genotypes with moderate resistance (Chartrain, Brading, Widdowson, & Brown, 2004) can be used in wheat breeding programs. Studies revealed that resistance follows the gene‐for‐gene model in the interaction between wheat genotypes and *Z. tritici* isolates (Brading et al., 2002; Kema et al., 2000; Kema et al., 2018).

In specific resistance, the avirulence gene (avir) of the pathogen is usually identified by the resistance gene (R) of the resistant genotype followed by induction of (HR) high resistance in the plant. Six isolates studied in this research were different in terms of high virulence to the studied genotypes. The RM 155 isolate was the most virulent isolate and should thus have fewer avirulence genes, on the contrary, the RM 251 isolate was the least virulent isolate and should have the most avirulence genes.

Long‐term cultivation of genotypes on a large scale may cause selection on the pathogenic population, thereby causing infection by dominating the resistance gene and breaking up the resistance. For example, resistance of genotypes containing *Stb1* and *Stb4* resistance genes in Argon was broken 5 years after its release due to evolution of the pathogen genotype (Adhikari et al., 2003; Chartrain, Brading, Makepeace, & Brown, 2004).

The research results revealed that the isolates had a different pathogenic pattern and were more virulence on most of the *Stb* genes, which were somewhat consistent with the findings of Abrinbana et al. (2012), Hosseinnezhad et al. (2014), and Mazandarani et al. (2014) and Makhdoomi et al. (2015). Studies around the world have indicated that *Stb* genes were vulnerable to attack by STB isolates (Abrinbana et al., 2012; Adhikari et al., 2003; Chartrain, Brading, Makepeace, & Brown, 2004; Cowger et al., 2000).

The present study also indicated that most of these *Stb* genes were not effective against Iranian *Z. tritici* isolates. This genetic diversity suggests that *Z. tritici* may be able to adjust quickly to resistant genotypes. Therefore, new sources of resistance must be regularly introduced to manage the disease. Using genetic resistance has been the most cost‐effective strategy for controlling this disease. Thus, identifying new sources of resistance and expanding wheat gene storage are essential for the management of this disease. According to the results of this study, *Stb1*, *Stb2*, *Stb3*, *Stb4*, *Stb5*, *Stb6*, *Stb7*, *Stb8*, *Stb9*, *Stb11*, *Stb13*, *Stb14*, and *stb18* genes were ineffective against the studied isolates, so they cannot be appropriate sources of resistance toward Iranian isolates of *Z. tritici*. The *Stb2*, *Stb5*, *Stb6*, *Stb7*, *Stb13*, and *Stb14* genes and *Stb2*, *Stb3*, *Stb4*, *Stb5*, *Stb6*, *Stb7*, *Stb8*, *Stb9*, *Stb11*, *Stb13*, *Stb14*, *Stb16*, *Stb17*, and *Stb18* were not effective against the Iranian isolates of *Z. tritici* according to the study of Hosseinnezhad et al. (2014) and Mahboubi et al. (2020), respectively. In this research, Shafir, Stanzuela Federal, and Courtot genotypes were susceptible against all isolates. Abrinbana et al. (2012) and Mahboubi et al. (2020) reported that Shafir (carry *Stb6* gene), Stanzuela Federal (carry *Stb7* gene), and Courtot (carry *Stb9* gene) genotypes were susceptible to all isolates. In this study, Flame (carry *Stb6* gene) (Brading et al., 2002) was resistant to all isolates. Similar results were obtained from Dalvand et al. (2016) research that Flame revealed resistance to Khuzestan isolate during 2013–2015. In the research of Kia et al. (2017), this genotype was resistant to two isolates. Simon et al. (2016) reported that the Flame indicated partial resistance to a higher number of fungal isolates. This genotype revealed specific resistance against three isolates in a study by Hosseinnezhad et al. (2014). Abrinbana et al. (2012) suggested that Flame genotype had specific resistance against six isolates. According to Makhdoomi et al. (2015), Flame was resistant to three isolates. The *Stb6*, *Stb7*, and *Stb11* genes have been identified in TE9111 genotype (Chartrain, Joaquim, et al., 2005). In this study, this genotype was also resistant to RM 251 and RM 155 isolates. In study of Tabib Ghaffary et al. (2012), this genotype was susceptible to one isolate, and in the other research, TE9111 was resistant to five isolates (Mahboubi et al., 2020). According to the study of Abrinbana et al. (2012), Hosseinnezhad et al. (2014), and Makhdoomi et al. (2015), this genotype indicated resistance against four, six, and three isolates, respectively. Arina and Riband genotypes were the source of resistance to STB, and have *Stb15* genes (Arraiano, 2007). Arina and Riband were resistant to all isolates in this study, but Riband had specific resistance against 12 isolates in the study of Hosseinnezhad et al. (2014) and was resistant to all isolates in a study by Makhdoomi et al. (2015). Arina genotype showed resistance to 6, 10, 18, 20, and 10 isolates in other researchers' studies (Mazandarani et al., 2014; Abrinbana et al., 2012; Hosseinnezhad et al., 2014; Mahboubi et al., 2020; Tabib Ghaffary et al., 2012). Czembor et al. (2011) studied the virulence spectrum of 23 fungal isolates of STB on differential genotypes and concluded that Arina was the most resistant genotype with *Stb15* genes, against European pathogenic isolates.

According to the results of this study, the Kavkaz‐K4500 genotype (carry *Stb10* and *Stb12* genes) was resistant to all isolates, which is consistent with the findings of Mahboubi et al. (2020). Rahnama and Rajabpour (2017) and Mohammad Beygi et al. (2014) reported that Kavkaz‐K4500 was susceptible to all isolates. Therefore, the genotypes Kavkaz‐K4500, Arina, and Riband have resistance genes toward Iranian isolates of *Z. tritici* that can be used as an effective resistance source in genotype breeding programs for STB resistance. Also, M3 which carries *Stb16* as well as *Stb17* was resistant to all isolates, this result was in agreement with the results of Mahboubi et al. (2020), Hosseinnezhad et al. (2014), and Tabib Ghaffary et al. (2012).

Estanzuela Federal, Israel493, Shafir, Courtot, Balance, M6 Synthetic (W‐7984), and Cs Synthetic 6x genotypes were susceptible to all isolates. These genotypes were also susceptible in previous research (kia et al., 2017; Mahboubi et al., 2020).

In this study, Salamouni (carrying *Stb13* and *Stb14*), Oasis (carrying *Stb1*), and Veranopolis (carrying *Stb2* and *Stb6*) genotypes have not been able to resist against RM 251, RM 5, RM 22, RM 183, and RM 230; RM 251, RM 5, RM 22, and RM 183; and RM 251 and RM 22 isolates, respectively.

Salamouni was resistant to eight and four isolates in the study of Mahboubi et al. (2020) and Dalvand et al. (2017), respectively. Oasis showed resistance to Iranian isolates during 2013–2015 (Dalvand et al., 2016). Also, Veranopolis resisted 13 and four isolates (Tabib Ghaffary et al., 2012; Mahboubi et al., 2020). So, it seems that the use of *Stb1*, *Stb2*, *Stb6*, *Stb13*, and *Stb14* genes cannot be effective in Iranian's breeding programs.

Tadinia (carrying *Stb4* and *Stb6*) and Bulgaria 88 (carrying *Stb1*) were resistant to RM 251 isolate. In the research of Dalvand et al. (2017), Tadinia among the foreign lines presented partial resistance to isolates, and Bulgaria 88 showed susceptibility to all the isolates. In studies on *Z. tritici* isolates by Abrinbana et al. and Makhdoomi et al. (2015), these genotypes were only resistant toward one or two isolates. This suggested that the *Stb1*, *Stb4*, and *Stb6* are ineffective against Iranian species, making them inefficient in wheat breeding programs.

The results of this study revealed that the tested isolates were virulent on most of the resistance genes and only a few genes had effective resistance against the isolates. This result was in agreement with previous reports (Abrinbana et al., 2012; Makhdoomi et al., 2015; Mehrabi et al., 2015).

*Stb1*, *Stb2*, *Stb4*, *Stb6*, *Stb7*, *Stb11*, *Stb13*, and *Stb14* genes were resistant to one or more isolates and the rest of the genes did not show resistance. Among the differential genotypes, Kavkaz‐K4500, Arina, Riband, Flame, and M3 genotypes had the highest resistance against of all the isolates. Therefore, they can be used in breeding programs to produce resistant genotypes to the disease.

## 5 CONCLUSIONS

The wheat landraces can be used successfully in wheat breeding programs to produce cultivars resistant to plant diseases. The other interesting result in our experiments was the high resistance of some wheat lines to most isolates, e.g., Dehdasht line was resistant to all isolates while landraces such as Shotordandan, Chamran 2, and Behrang were semisensitive. Therefore, it is likely a novel resistance gene(s) to *M. graminicola* isolate in these lines. In addition, most of these lines may have had several resistance genes with different effects, resulting in a high resistance to various isolates in general.

Iran has been considered as the center of diversity wheat and *M. graminicola* fungi (Stukenbrock et al., 2007) and the pathogen and host plant have been in continuous interaction and evolution for thousands of years, so the Iranian isolates of *M. graminicola* with high genetic diversity and various virulence spectrum can dominate most of the known *Stb* genes. Also, they can be used to investigate resistance of wheat genotypes toward STB in other important wheat cultivation areas of the country, especially in Golestan, Khuzestan, and Ardebil provinces. Moreover, it is required to continuously study genetic changes in the pathogenic fungal population and to identify genetic sources of resistance with effective resistance genes against pathogenic isolates.

## AUTHOR CONTRIBUTIONS

**Tayebeh Bakhshi:** Data curation (equal); formal analysis (equal); investigation (equal); methodology (equal); software (equal); writing – review and editing (lead). **Farajollah Shahriari Ahmadi:** Project administration (equal). **Mostafa Aghaee Sarbarzeh:** Project administration (lead). **Rahim Mehrabi:** Project administration (equal). **Alireza Seifi:** Supervision (equal).

## CONFLICT OF INTEREST STATEMENT

The authors claim no conflict and interests in this work.

## ETHICS STATEMENT

All procedures performed in studies did not involve human participants and animals.

## Data Availability

Author elects to not share data.

## References

[fsn33578-bib-0044] Abrinbana, M. , Makhdoomi, A. , Mehrabi, R. , & Khodarahmi, M. (2015). Efficacy of wheat genotypes and *Stb* resistance genes against Iranian isolates of *Zymoseptoria tritici* . Journal of General Plant Pathology, 81, 5–14. 10.1007/s10327-014-0565-8

[fsn33578-bib-0001] Abrinbana, M. , Mozafari, J. , Shams‐bakhsh, M. , & Mehrabi, R. (2010). Genetic structure of Mycosphaerella *graminicola* populations in Iran. Plant Pathology, 59, 829–838. 10.1111/j.1365-3059.2010.02309.x

[fsn33578-bib-0002] Abrinbana, M. , Mozafari, J. , Shams‐bakhsh, M. , & Mehrabi, R. (2012). Resistance spectra of wheat genotypes and virulence pattern of *Mycosphaerella graminicola* isolates in Iran. Euphytica, 186, 75–90. 10.1007/s10681-011-0493-z

[fsn33578-bib-0003] Adhikari, T. B. , Anderson, J. M. , & Goodwin, S. B. (2003). Identification and molecular mapping of a gene in wheat conferring resistance to *Mycosphaerella graminicola* . Phytopathology, 93, 1158–1164. 10.1094/Phyto.2003.93.9.1158 18944101

[fsn33578-bib-0004] Adhikari, T. B. , Cavaletto, J. R. , Dubcovsky, J. , Gieco, J. O. , Schlatter, A. R. , & Goodwin, S. B. (2004). Molecular mapping of the *Stb4* gene for resistance to septoria rtitici blotch in wheat. Phytopathology, 94, 1198–1206. 10.1094/Phyto.2004.94.11.1198 18944455

[fsn33578-bib-0005] Adhikari, T. B. , Wallwork, H. , & Goodwin, S. B. (2004). Microsatellite markers linkes to the *Stb2* and *Stb3* genes for resistance to septoria tritici blotch in wheat. Crop Science, 44, 1403–1411. 10.2135/cropsci2004.1403

[fsn33578-bib-0006] Adhikari, T. B. , Yang, X. , Cavaletto, J. R. , Hu, X. , Buechley, G. , Ohm, H. W. , Shaner, G. , & Goodwin, S. B. (2004). Molecular mapping of Stb1, a potentially durable gene for resistance to septoria tritici blotch in wheat. Theoretical and Applied Genetics, 109, 944–995. 10.1007/s00122-004-17096 15490099

[fsn33578-bib-0007] Arraiano, L. S. , Chartrain, L. , Bossolini, E. , Slatter, H. N. , Keller, B. , & Brown, J. K. M. (2007). A gene in European wheat cultivars for resistance to an African isolate of Mycosphaerella graminicola. Plant Pathology, 56(1), 73–78. 10.1111/j.1365-3059.2006.01499.x

[fsn33578-bib-0008] Arraiano, L. S. , Worland, A. J. , Ellerbrook, C. , & Brown, J. K. M. (2001). Chromosomal location of a gene for resistance to septoria tritici blotch (*Mycosphaerella graminicola*) in the hexaploid wheat ‘synthetic 6x’. Theoretical and Applied Genetics, 103, 758–776. 10.1007/s001220100668

[fsn33578-bib-0009] Bashiri, A. , Torabi, M. , & Dadrezaie, S. T. (2006). Investigation of pathogenic differences between pathogenic fungal isolates wheat leaf septoria in Iran. In Abstracts of the 17th Iranian plant protection congress (p. 8). University of Tehran.

[fsn33578-bib-0010] Brading, P. A. , Verstappen, E. C. P. , Kema, G. H. J. , & Brown, J. K. M. (2002). A gene_ for _ gene relationship between wheat and *Mycosphaerella graminicola*, the septoria tritici blotch pathogen. Phytopathology, 92, 439–445. 10.1094/Phyto.2002.92.4.439 18942957

[fsn33578-bib-0011] Brown, J. K. M. , Kema, G. H. J. , Forrer, H. R. , Verstappen, E. C. P. , Arraiano, L. S. , Brading, P. A. , Foster, E. M. , Fried, P. M. , & Jenny, E. (2001). Resistance of wheat cultivar and breeding lines to Septoria tritici blotch caused by *Mycosphaerella graminicola* in field trials. Plant Pathology, 50, 325–338. 10.1046/j.1365-3059.2001.00565.x

[fsn33578-bib-0012] Chartrain, L. , Berry, S. T. , & Brown, J. K. M. (2005). Resistance of wheat line Kavkaz‐K4500 L.6.A.4 to Septoria tritici blotch controlled by isolate specific resistance genes. Phytopathology, 95, 664–671. 10.1094/Phyto-95-0664 18943783

[fsn33578-bib-0013] Chartrain, L. , Brading, P. A. , & Brown, J. K. M. (2005). Presence of the Stb6 gene for resistance to septoria tritici blotch (Mycosphaerella graminicola) in cultivars used in wheat‐breeding programmes worldwide. Plant Pathology, 54, 134–143. 10.1111/j.1365-3059.2005.01164.x

[fsn33578-bib-0014] Chartrain, L. , Brading, P. A. , Makepeace, J. C. , & Brown, J. K. M. (2004). Sources resistance to Septoria tritici and implication for wheat breeding. Plant Pathology, 53, 454–460.

[fsn33578-bib-0015] Chartrain, L. , Brading, P. A. , Widdowson, J. P. , & Brown, J. K. M. (2004). Partial resistance to Septoria tritici blotch *(Mycosphaella graminicola)* in wheat cultivar Arina and riband. Phytophatology, 94, 497–504. 10.1046/j.0032-0862.2004.01052.x 18943769

[fsn33578-bib-0016] Chartrain, L. , Joaquim, P. , Berry, S. T. , Arraiano, L. S. , Azanza, F. , & Brown, J. K. M. (2005). Genetics of resistance to *Septoria tritici* blotch in the Portuguese wheat breeding line TE 9111. Theoritical and Applied Genetics, 110, 1138–1144. 10.1007/s00122-005-1945-4 15759105

[fsn33578-bib-0017] Chartrain, L. , Sourille, P. , Bernard, M. , & Brown, J. K. M. (2009). Identification and location of *Stb9*, a gene for resistance to septoria tritici blotch in wheat cultivars Courtot and tonic. Plant Pathology, 58, 547–555. 10.1111/j.1365-3059.2008.02013.x

[fsn33578-bib-0018] Cowger, C. , Hoffer, M. J. L. , & Mundt, C. C. (2000). Specific adaptation by *Mycosphaerella graminicola* to a resistant wheat cultivar. Plant Pathology, 49, 445–451. 10.1046/j.1365-3059.2000.00472.x

[fsn33578-bib-0019] Cowling, S. G. (2006). Identification and mapping of host resistance genes to Septoria tritici blotch of wheat. PhD Thesis. University of Manitoba.

[fsn33578-bib-0020] Czembor, P. C. , Radecka‐Janusik, M. , & Mankowski, D. (2011). Virulence spectrum of *Mycosphaerella graminicola* isolates on wheat genotypes carrying known resistance genes to Septoria tritici blotch. Journal of Phytopathology, 159, 146–154. 10.1111/j.1439-0434.2010.01734.x

[fsn33578-bib-0021] Dadrezaie, S. T. , Minassian, V. , Torabi, M. , & Lotfali, G. (2003). Effect of Septoria tritici infection at different growth stages on yield components of three wheat cultivars. Seed and Plant, 19, 101–116.

[fsn33578-bib-0022] Dalvand, M. , Soleimani Pari, M. J. , & Zafari, D. (2017). Evaluating the efficacy of STB resistance genes to Iranian *Zymoseptoria tritici* isolates. Journal of Plant Diseases and Protection, 125(1), 27–32. 10.1007/s41348-017-0143-3

[fsn33578-bib-0023] Dalvand, M. , Soleimani Pari, M. J. , Zafari, D. , Roohparvar, R. , & Tabib Ghaffari, S. M. (2016). Study on virulence factors of *Mycosphaerella graminicola*, the causal agent of Septoria leaf blotch and reactions of some Iranian wheat genotypes to this pathogen in Iran. Journal of Applied Biotechnology Reports, 3, 359–363.

[fsn33578-bib-0024] Davari, M. , Abrinbana, M. , Asghari Zakaria, R. , & Arzanlou, M. (2012). Assesment of wheat cultivars for resistance to *Mycosphaerella graminicola* isolates from Moghan plain at seedling stage under greenhouse conditions. Iranian Journal of Plant Protection Science, 43, 379–383. 10.22059/IJPPS.2013.30257

[fsn33578-bib-0025] Desmaziers, J. B. H. J. (1842). Neuvieme notice sur quelques plantes cryptogames. Annales des science naturelles, Serie Botanique, 2(1842), 91–118.

[fsn33578-bib-0026] Eyal, Z. (1999). The septoria tritici and stagonospora nodorum blotch disease of wheat. European Journal of Plant Pathology, 105, 629–641. 10.1023/A:1008716812259

[fsn33578-bib-0027] Eyal, Z. , Amiri, Z. , & Wahl, I. (1973). Physiologic specialization of septoria tritici. Phytopathology, 63, 1087–1091. 10.1094/Phyto-63-1087

[fsn33578-bib-0028] Eyal, Z. , Scharen, A. L. , Prescott, J. M. , huffman, M. D. , & Van Ginkel, M. (1985). Global insights into virulence frequencies of *Mycosphaerella graminicola* . Phytopathology, 75, 1456–1462. 10.1094/Phyto-75-1456

[fsn33578-bib-0029] Eyal, Z. , Scharen, A. L. , Prescott, J. M. , & Van Ginkel, M. (1987). The septoria disease of wheat. Consepts and methods of disease management. CYMMYT.

[fsn33578-bib-0030] Ghaneie, A. , Mehrabi, R. , Safaie, N. , Abrinbana, M. , saidi, A. , & Aghaee, M. (2012). Genetic variation for resistance to septoria tritici blotch in Iranian tetraploid wheat landraces. European Journal of Plant Pathology, 132, 191–202. 10.1007/s10658-011-9862-7

[fsn33578-bib-0031] Grieger, A. , Lamari, L. , & Brule‐Babel, A. (2005). Physiologic variation in Mycosphaerella graminicola from western Canada. Canadian Journal of Plant Pathology, 27, 71–77. 10.1080/07060660509507196

[fsn33578-bib-0032] Habibi, M. , Mirakhorli, N. , Shiran, B. , & Mardi, M. (2014). Study of resistance related gene expression pattern to septoria tritici blotch (STB) in wheat (*Triticum aestivum*). Modern Gen., 8(2), 149–158.

[fsn33578-bib-0033] Haghdel, M. , & Banihashemi, Z. (2003). Reaction of wheat cultivars to isolate of septoria tritici under greenhouse and control chamber conditions. Iranian Journal of Plant Pathology, 39, 175–187.

[fsn33578-bib-0034] Hoorne, C. , Lamari, J. , gilbert, J. , & Balance, G. M. (2002). First report of *Mycosphaerella graminicola,* the sexual state of septoria tritici in Manitoba Canada. Plant Pathology, 24, 445–449. 10.1080/07060660209507032

[fsn33578-bib-0035] Hosseinnezhad, A. , Khodarahmi, M. , Rezaee, S. , Mehrabi, R. , & Roohparvar, R. (2014). Effectiveness determination of wheat genotypes and *Stb* resistance genes against Iranian *Mycosphaerella graminicola* isolates. Arch Phytopathol Plant Prot, 47, 2051–2069. 10.1080/03235408.2013.868696

[fsn33578-bib-0036] Kema, C. H. J. , Verstappen, E. C. P. , & Waalwijk, G. (2000). A virulence in the wheat *Septoria tritici* leaf blotch fungus *Mycosphaerella graminicola* is controlled by a single locus. Molecular Plant‐Microbe Interactions, 13, 1375–1379. 10.1094/MPMI.2000.13.12.1375 11106030

[fsn33578-bib-0037] Kema, G. H. J. , Annane, J. G. , Sayoud, R. , Van Silfhout, C. H. , Van Ginkel, M. , & Debree, J. (1996). Genetic variation for virulence and resistance in the wheat *Mycosphaerella graminicola* pathosystem interactions between pathogen isolates and host cultivars. Phytopathology, 86, 200–212. 10.1094/Phyto-86-200 18945169

[fsn33578-bib-0038] Kema, G. H. J. , Gohari, A. M. , Aouini, L. , Gibriel, H. A. , Ware, S. B. , Van Den Bosch, F. , Manning‐Smith, R. , Alonso‐Chavez, V. , Helps, J. , M'Barek, S. B. , Mehrabi, R. , Diaz‐Trujillo, C. , Zamani, E. , Schouten, H. J. , Van Der Lee, T. A. J. , Waalwijk, C. , De Waard, M. A. , De Wit, P. J. G. M. , Verstappen, E. C. P. , … Seidl, M. F. (2018). Stress and sexual reproduction affect the dynamics of the wheat pathogen effector *AvrStb6* and strobilurin resistance. Nature Genetics, 50, 375–380. 10.1038/s41588-018-0052-9 29434356

[fsn33578-bib-0039] Kema, G. H. J. , Verstappen, E. C. P. , Todorova, M. , & Waalwijk, C. (1996). Successful crosses and molecular tetrad and progeny analyses demonstrate heterothallism in *Mycosphaerella graminicola* . Current Genetics, 30, 251–258. 10.1007/s002940050129 8753655

[fsn33578-bib-0040] Khelghatibana, F. , Dadrezaie, S. T. , Dehghan, M. A. , Nazari, K. , & Torabi, M. (2004). Responses of eleven commercial wheat cultivar to septoria tritici in field. Proceeding of the 16th Iranian Plant Protection Congress, Tabriz, p. 11.

[fsn33578-bib-0041] Kia, S. , Rahnama, K. , Soltanloo, H. , Babaeizad, V. , & Aghajani, M. A. (2017). Effectiveness of resistance genes to *Septoria tritici* blotch (*Stb*) in differential Cultivares of wheat against *Zymoseptoria tritici* isolates. Applied Research in Phytomedicine, 6, 3.

[fsn33578-bib-0042] Kia, S. , Torabi, M. , & Nazari, K. (2006). Evaluation of resistance to septoria leaf blotch in wheat lines and cultivars. Proceedings of the 17th Iranian Plant Protection Congress, Karaj. P., 3.

[fsn33578-bib-0043] Mahboubi, M. , Talebi, R. , Aghaee Sarbarzeh, M. , Naji, A. M. , & Mehrabi, R. (2020). Resistance and virulence variability in wheat *Zymoseptoria tritici* interactions. Crop & Pasture Science, 71(7), 645–652. 10.1071/CP20126

[fsn33578-bib-0045] Makhdoomi, M. A. , Mehrabi, R. , Khodarahmi, M. , Bihamta, M. , & Zad, S. J. (2011). Study of the reaction of some commercial cultivars and advanced wheat lines to septal isolates (Septoria tritici) leaf. Journal of Agriculture and Plant Breeding, 7(1), 2–12.

[fsn33578-bib-0046] Mazandarani, T. , Mehrabi, R. , & Maleki, M. (2014). Efficacy of Stem's leaf spot resistance (Stb) resistance genes against isolates of Mycosphaerella graminicola in Fars province. Seed and Plant Journal, 1–30(3), 6–669.

[fsn33578-bib-0047] McCartney, C. A. , Brule‐Babel, A. L. , Lamari, L. , & Somers, D. J. (2003). Choromosomal location of a race‐specific resistance gene to *Mycosphaerella graminicola* in the spring wheat ST6. Theoretical Applied Genetics, 107, 1181–1186. 10.1007/s00122-003-1359-0 12898022

[fsn33578-bib-0048] Mehrabi, R. , Makhdoomi, A. , & Jafar‐Aghaee, M. (2015). Identification of new sources of resistance to septoria tritici blotch caused by *Zymoseptoria tritici* . Journal of Phytopathology, 163, 84–89. 10.1111/jph.12282

[fsn33578-bib-0049] Mergoum, M. , Singh, P. K. , Ali, S. , Elias, E. M. , Anderson, J. A. , Glover, K. D. , & Adhikari, T. B. (2007). Reaction of elite wheat genotypes from northern great plains to septoria diseases. Plant Disease, 91, 1310–1315. 10.1094/PDIS-91-10-1310 30780524

[fsn33578-bib-0050] Mohammad Beygi, A. , Roohparvar, R. , & Torabi, R. (2014). Pathogenicity variation in isolates of *Mycosphaerella graminicola* the Septoria tritici blotch pathogen on differential cultivars. Seed and Plant Journal, 2, 31. 10.22092/SPIJ.2017.111259

[fsn33578-bib-0051] Quaedvlieg, W. , Kema, G. H. J. , Groenewald, J. Z. , Verkley, S. , Seifbarghi, G. J. M. , Razavi, M. , Mirzadi Gohari, A. , Mehrabi, R. , & Crous, P. W. (2011). Zymoseptoria gen. Nov: A new genus to accommodate Septoria‐like species occurring on *graminicolous* hosts. Persoonia, 98, 57–69. 10.3767/003158511X571841 PMC3160802 22025804

[fsn33578-bib-0052] Rahnama, H. , & Rajabpour, S. (2017). Factors for Consumer Choice of Dairy Products in Iran. Appetite, 111, 46–55. 10.1016/j.appet.2016.12.004 27988369

[fsn33578-bib-0053] Rajaie, S. , Dabbagh, G. , & Noorollahi, K. H. (2004). Study on the effect of some systemic fungicides against septoria leaf blotch of wheat. Proceeding of the 16th Iranian Plant Protection Congress, Tabriz. P. 15.

[fsn33578-bib-0054] Razavi, M. , & Hughes, G. R. (2003). Pathogenic and molecular variability in a population of Mycosphaerella graminicola, cause of Septoria leaf blotch of wheat. Ph. D. thesis. University of Saskatchewan.

[fsn33578-bib-0055] Rosielle, A. A. (1972). Sources of resistance in wheat to speckled leaf blotch caused by septoria tritici. Euphytica, 21, 152–161. 10.1007/BF00040560

[fsn33578-bib-0056] Sanderson, F. R. (1972). A *Mycosphaerlla* species as the ascogenous state of septoria tritici. Rob. Ex. Desm. New Zealand Journal of Botany, 10, 707–709. 10.1139/B78-084

[fsn33578-bib-0057] Shearer, B. L. , & Wilcoxson, R. D. (1978). Variation in the size of macropycnidiospores and pycnidia of septoria tritici on wheat. Botany, 56, 742–746.

[fsn33578-bib-0058] Simon, M. R. , Castillo, N. S. , & Cordo, C. A. (2016). New sources of resistance to *Septoria tritici* blotch in wheat seedlings. European Journal of Plant Pathology, 146, 625–635. 10.1023/B:EUPH.0000047059.57839.98

[fsn33578-bib-0059] Somasco, O. A. , Qualset, C. O. , & Gilchrist, D. G. (1996). Single‐gene resistance to Septoria tritici blotch in the spring wheat cultivar Tadinia. Plant Breeding, 115, 261–267.

[fsn33578-bib-0060] Stukenbrock, E. H. , Banke, S. , Javan‐Nikkhah, M. , & McDonald, B. A. (2007). Origin and domestication of the fungal wheat pathogen *Mycosphaerella graminicola* via sympatric speciation. Molecular Biology and Evolution, 24, 398–411. 10.1093/molbev/msl169 17095534

[fsn33578-bib-0061] Tabib Ghaffary, S. M. , Faris, J. D. , Friesen, T. L. , Visser, R. G. , Van Der Lee, T. A. , Robert, O. , & Kema, G. H. (2012). New broad‐spectrum resistance to *Septoria tritici* blotch derived from synthetic hexaploid wheat. Theoretical and Applied Genetics, 124, 125–142. 10.1007/s00122-011-1692-7 21912855 PMC3249545

[fsn33578-bib-0062] Tabib Ghaffary, S. M. , Robert, O. , Laurent, V. , Lonnet, P. , Margale, E. , Van Der Lee, T. A. J. , Visser, R. G. F. , & Kema, G. H. J. (2011). Genetic analysis of resistance to septoria tritici blotch in the French winter wheat cultivars Balance and apache. Theoretical and Applied Genetics, 123, 741–775. 10.1007/s00122-011-1623-7 21655994 PMC3155673

[fsn33578-bib-0063] Van Ginkel, M. , & Schafren, A. L. (1988). Host‐pathogen relationships of wheat and septoria tritici. Phytopathology, 78, 762–766. 10.1094/Phyto-78-762

[fsn33578-bib-0064] Yang, N. , McDonald, M. C. , Solomon, P. S. , & Milgate, A. W. (2018). Genetic mapping of *Stb19*, a new resistance gene to *Zymoseptoria tritici* in wheat. Theoretical and Applied Genetics, 131, 2765–2773. 10.1007/s00122-018-3189-0 30238255

